# Supplementary material for: Connectivity concepts in neuronal network modeling
Source: PLoS Comput Biol. 2022 Sep 8;18(9):e1010086. doi: 10.1371/journal.pcbi.1010086 (PMC9455883; doi:10.1371/journal.pcbi.1010086)
Supplement: Attachment — Submitted filename: Senk_Connectivity_rev1_response.pdf [file pcbi.1010086.s001.pdf]

Jülich, 4 February 2022

Dear Prof. Toro, dear Prof. Marinazzo,

Thank you very much for the opportunity to revise our manuscript "Connectivity Concepts in Neuronal Network Modeling" as suggested in your email from 14 December 2021. Please find enclosed our detailed responses to the points raised by the reviewers together with the revised manuscript. Changes compared to the previous submission are shown in blue with a reference to the corresponding reviewer's point as a superscript.

Our responses include

- remarks on network sizes and scaling;
- confinement of considerations on technical implementations to the Discussion;
- a table summarizing connection methods replacing their textual description;
- an elaboration on our use of the terms "devices" and "bidirectional";
- explicit encouragement for the researcher to extend and customize the graphical notation on the examples of excitation and inhibition as well as gap junctions;
- a list of all articles included in the literature review; and
- improvement of Fig 3B (Venn diagram with layout now informed by the data) and Fig 8C (distinction between concepts and explicit parameters).

Besides, we have added the Helmholtz Metadata Collaboration (HMC) as a further funding source.

We are very grateful to the two reviewers for their detailed reports, which helped us to considerably improve the manuscript. We are confident that the revised manuscript meets the high standards requested by PLOS CB.

With kind regards,

Johanna Senk, on behalf of the authors.

Reviewer 1

Reviewer: *This manuscript outlines a set of concepts, expressions and graphical entities which can be used to make network connectivity in models in computational neuroscience more explicit, reproducible and sharable. The ideas build on existing concepts used in simulators and published models and develops past work in this area by a number of the authors. The manuscript is well written and the concepts clearly presented, and can potentially form the basis of a consensus community approach to expressing and sharing such network models. The manuscript, while long, has no major flaws, but a few minor changes would improve it.*

We thank the reviewer for the positive assessment of our work.

Reviewer (R1P1): *It would be good to have an explicit list (in an appendix perhaps) of the 42 network models considered in the "Networks used in the computational neuroscience community" section.*

As suggested by the reviewer, we have compiled a list of all reviewed articles. This list is included in the revised version of the manuscript in the Section "Materials and methods".

Reviewer (R1P2): *A bit more should be said about "devices" when they are introduced on page 22.*

We have added an explanatory sentence at the location indicated by the reviewer. We define devices as network nodes that provide an external stimulus (e.g., a current input) to the main neuronal network or record simulated network activity.

Reviewer (R1P3): *Re bold and plain outlines for Node classes - surely there is no need for making this distinction/requirement if there is only one class of node, i.e. either single units or just populations. The description here could be clarified.*

We see it in the same way as the reviewer: assigning meaning to the outlines only makes sense if the diagram requires the distinction of different node classes. We would like to kindly point the reviewer to the sentence in line 812f of the original submission saying: "The distinction is primarily a recommendation for diagrams that contain both kinds of nodes." To emphasize this argument, we have now removed the word "primarily".

Reviewer (R1P4): *There should be more discussion on what precisely defines an "excitatory" or "inhibitory" node. For simple neuronal models this is clear, but for more biophysically realistic circuits, glutamatergic or GABAergic synapses can act in the opposite way to the usual depending on the membrane potential of the post synaptic cell.*

The reviewer addresses the important point that the proposed graphical notation should be considered as a starting point. Several definitions are kept general on purpose to leave it up to the researcher to apply the generic graphical elements to specific models and add details if necessary. In particular, the common but often too simple classification of neural nodes into excitatory and inhibitory types

should be adapted if required. We have elaborated on this remark by the reviewer at the end of Section "Customization and extension" to provide an example of how the notation can develop further. Section "Cortical microcircuit with distinct interneuron types" already takes up the topic by making a suggestion for representing different inhibitory types.

Reviewer (R1P5): *More should be said about electrically coupled networks containing gap junctions. These are often incorporated in networks even with single compartment cells, and it would just be a small amount of work to extend the discussion/graphical notation to incorporate these. They are often depicted as symmetric connections with flat/T shaped arrowheads on each end.*

The reviewer is right that the graphical notation so far does not include gap junctions but that they are relevant for many neuronal network models. The revised manuscript addresses this point as follows: At the end of Section "Customization and extension", we propose gap junctions as a novel edge type and use this as an example of how to extend the basic definitions of the notation.

However, we have decided to represent the gap junctions with a zig-zag line as used, for instance, by [1]. The suggested flat/T shaped arrowheads have been used occasionally for inhibitory connections and SBGN also uses them with this meaning, see:

`https://sbgn.github.io/symbols#pd-inhibition`
With our choice, we hope to avoid confusion.

Reviewer (R1P6): *It should be clarified when Bidirectional connections are mentioned that in the case that there are 2 populations A&B with connections A→B and B→A, that 2 independent connections/arrows should be depicted (presumably with different parameters/conditions for each direction).*

As suggested by the reviewer, we have clarified our definition of "Bidirectional" connections at the end of the respective paragraph of the manuscript. We propose to use the bidirectional arrow with arrowheads on both ends only if both the reciprocally connected units are identical as well as all parameters of the connections. If connections are not symmetrical in that sense, we advise to use two arrows.

Reviewer (R1P7): *At line 961: autapses are prohibited and multapses allowed - therefore no M with line through it required here.*

We thank the reviewer for being so careful. The mistake has been corrected.

Reviewer (R1P8): *In Fig 8A, it is a bit difficult to see why Kin,EE, Kin,IE etc. aren't used in this depiction as opposed to just Kin 4 times, since 4 independent connections are shown. Also, the rest of the text implies Kin,EE and Kin,IE are independent values, whereas panel E suggests a single value K_in_TE is used for E → E and E → I.*

We agree with the reviewer that Fig 8 and its description did not clearly distinguish between concepts and explicit parameters. The symbols in the network diagram in panel A refer to the concepts, for example K_{in} represents the rule "random, fixed in-degree". With further subscripts, parameters could be made explicit in a diagram, but here we have decided to focus on the concepts to avoid clutter.

In the other panels, $K_{\mathrm{in},\mathcal{T}\mathrm{E}}$ and $K_{\mathrm{in},\mathcal{T}\mathrm{I}}$ represent the explicit values used for the in-degrees. We have clarified this distinction in the figure caption.

In addition, the table in panel C now also uses $K_{\mathrm{in},\mathcal{T}\mathrm{E}}$ and $K_{\mathrm{in},\mathcal{T}\mathrm{I}}$ without explicitly resolving the target population $\mathcal{T} \in \{\mathrm{E}, \mathrm{I}\}$ because the in-degree values in the network model used are in fact independent of the targets.

Reviewer 2

Reviewer: *Authors propose a methodology to fix a well known problem in computational neuroscience related to the (generally) ambiguous description of connectivity in neural models.*

We appreciate that the reviewer recognizes the relevance of the problem tackled by our manuscript.

Reviewer (R2P1): *Starting from an illustrative example, authors first review some terminology that is widely used in the computational neuroscience community, emphasizing and justifying the common usage of high level description for connectivity patterns. The introduction cites a lot of different studies to highlight the richness of connectivity patterns but I don't think this is really necessary (i.e. it is a well known fact). Authors should instead go straight to the question they're interested to answer without giving too broad considerations. For example, the paragraph on exascale is superfluous and I don't understand the link between a faithful description of connectivity and the ease of implementation at this stage.*

We agree with the reviewer that the paragraph on novel and future computing systems was insufficiently motivated in the Introduction. Therefore, we have moved it to the Discussion (slightly modified) where we relate the description of connectivity concepts to their implementation. Considering the richness of connectivity patterns a well-known fact that does not require further emphasis might be true for the experienced neuroscientist. But for completeness and reference in particular for researchers from related scientific domains, we still find it useful to give an insight into the neuroanatomy. As the target audience of this article, we consider both computational neuroscientists and developers of simulation technology such as neuromorphic hardware, who might be not that familiar with constraints posed by nature.

Reviewer (R2P2): *Furthermore, one important concept that may need to be introduced concerns the scalability of models: if your connectivity description is precise enough, you should be able to build a model with n=100 or n=1000 without much change in the results.*

The scalability of models is indeed an important point which the revised manuscript addresses in the Introduction: We have added orders of magnitude for neuron and synapse numbers in the natural-density, full-scale cortical network for reference (i.e., 10^{10} neurons and 10^{14} connections in human cortex) and emphasized that only some but not all effects on the neuronal dynamics can be compensated for when downscaling a network [2]. Both neuron numbers given by the reviewer, $n = 100$ and $n = 1000$, are in this regard small compared to the cortical network. The connectivity concepts considered in the study are in general independent of the network size and can be applied to both small and large networks; we have expanded on this when defining the scope of the study. However, an explicit analysis of finite size effects for the derived distributions goes beyond the present study.

Reviewer (R2P3): *The introduction is followed by a small but detailed literature review (n=42) that exhibits the diversity of practices in both manuscripts and codes. While this literature is really interesting, I think it would deserve to be shorten since I think some data are not really relevant. For example, I don't think figure 2 brings anything useful for the debate and is also bit difficult to read, especially the Venn diagram that brings more confusion than it helps. These Venn diagrams are also*

used in other figures and bring equal confusion. For example, figure 3B is unreadable. Overall, I read this section as a detailed sequential description of the review results (which again are really good), but I would have expected a shorter and structured overview of practices and have put the actual details in the supplementary materials.

We appreciate that the reviewer values the literature review and its results.

While we see the point that this review is lengthy, we are hesitant to move parts of it into supplementary materials. We find it crucial to provide sufficient information about the origin of the data in the main paper for the reader to be able to judge the representativeness of the results including the recency of the studies analyzed.

We agree that the Venn diagram of Fig 3B was confusing; it was a general setup which in theory is capable of showing all possible relations between any four sets but in practice hard to read. The revised figure panel is informed by the data and makes use of the observation that the set "Text" intersects with all the other sets: This fact allows us to draw a Venn diagram of only three sets inside of a circle depicting "Text". We hope that the reviewer finds this representation clearer.

As an alternative for Venn diagrams we have tested UpSet plots [3]. But in the end, we have decided to keep Venn diagrams throughout for intersecting data sets because the number of sets to be shown is always small (the maximum four in Fig 3B has just been simplified) and we would like to avoid introducing yet another diagram style even more readers might not be familiar with.

Reviewer (R2P4): *The section on languages and simulators is structured around procedural descriptions, declarative population-level descriptions, algebraic descriptions followed by a specific subsection about simulators and the different methods this used. This again a very detailed description (with even the name of methods for each simulator) that could be summarized withe a simple table and whether they implements this or that specific feature. As it is written, I find really hard to know which simulator does what.*

We have revised the end of Section "Population-level connectivity rules of languages and simulators" following the constructive suggestion of the reviewer: To convey a better overview of which rules are available, we now provide more concise text supported by a summarizing table.

Reviewer (R2P5): *The connectivity concepts section is clear and well structured, I do not have much comments but one on the subsection on network embedded in metric spaces. I wonder why authors do not generalize this specific case by considering the case where a function f: S (sources) x T (targets) -¿ R (reals) exists and could be used for defining the connectivity. If your network is embedded in a metric space, you have a "natural" function f with any distance function, but I can imagine cases where you do not have such metrics and yet there are specific relation between units (through the function f).*

We thank the reviewer for this comment and have extended the Section "Networks embedded in metric spaces" to also suggest this generalization.

Reviewer (R2P6): *Based on these connection concepts, authors proposed a graphical notation to specify connectivity in a non ambiguous manner. While I appreciate the effort, I'm not totally convinced by the different examples that are given because I think there are really two different kinds of readers to consider. For example, figure 10 shows the patchy connection. On the one hand, figure 10b allows me to immediately get a sense of the connectivity pattern even though there is not enough*

information to implement it. On the other hand, figure 10c allows me to implement the patchy connection, but I do not get a sense of what it actually "looks like". In the end, I feel we may need both: approximate graphical representations for humans, and precise graphical representations for computers. Consequently, it would be worth to add an advice from the authors on when and how to use such graphical representations in an article.

We strongly agree with the reviewer that different representations for humans and computers are needed. All network diagrams shown in our manuscript are intended for humans, and only toward the end of the Discussion, we address implications for implementation. With Figs 9 and 10 we aim to demonstrate that multiple diagrams showing the network model on different levels of detail can be combined, in particular for complex and hierarchical networks. In this way, the modeler can complementarily provide the reader with an intuitive overview and precise details for reproducibility.

Reviewer (R2P7): *I've also one small question on the necessity of having a specific representation of inhibitory neurons knowing that you also have a specific representation for inhibitory edge. What would be the difference between an excitatory source node sending a inhibitory edge and an inhibitory source node sending an inhibitory edge?*

In the basic definition we provide, excitation and inhibition are per default encoded redundantly in both neuron and edge type to aid intuition. However, we do not explicitly prohibit the above detailed case of an inhibitory edge originating from an excitatory neuron – assuming that the modeler has a good reason for that choice. Please refer also to our response to point R1P4, which encourages researchers to go beyond the basic definitions and add more model-specific detail if required.

Reviewer: *Overall, I think this article is important and is worth to be published but I would advised authors to either drastically restructure/shorten (in order to focus on the main proposal) or to split the article in two articles (I & II in the same journal). The reason is that I think there are really two articles in one. One is about a review of practices in computational neurosciences based on a literature review and simulators implementation, with the various implications in term of reproducibility. This is already an interesting review and could be complemented by a state of the art in that domain. The second article is precisely a proposal for a generic and non-ambiguous connectivity description, supported by several examples and could be further extended with supplementary figures (for example, all the variation of an ambiguous textual description). Note that I'm not asking for such additions in the present article but only if there were two different articles. Finally, I suggested some modifications in the manuscript in hope it would improve it but I'm perfectly fine if authors prefer not to perform such modifications as long as they explain why they prefer not to change.*

We appreciate that the reviewer finds the manuscript worth publishing and we have implemented most of the suggestions for improvement. After serious consideration, however, we came to the conclusion that we do not want to drastically shorten or split the article into two as proposed by the reviewer, and we have a great regard for the reviewer to leave this final decision up to us. We see the argument that the manuscript is quite comprehensive and combines two different aspects: a literature review and novel proposals. In the case of our manuscript, these two parts are tightly connected as the former is the basis for the latter; the latter is to a large extent derived directly from the former. For the benefit of the reader, we therefore find it useful to have the snapshot in time of how the community handles connectivity closely together with the prospect of how connectivity descriptions could be improved in the future. Overall, we thank the reviewer for the detailed and considerate comments.

References

[1] Velazquez JLP, Carlen PL. Gap junctions, synchrony and seizures. Trends in Neurosciences. 2000 Feb;23(2):68–74. doi:10.1016/s0166-2236(99)01497-6.

[2] van Albada SJ, Helias M, Diesmann M. Scalability of Asynchronous Networks Is Limited by One-to-One Mapping between Effective Connectivity and Correlations. 2015 Sep;11(9):e1004490. doi:10.1371/journal.pcbi.1004490.

[3] Lex A, Gehlenborg N, Strobelt H, Vuillemot R, Pfister H. UpSet: Visualization of Intersecting Sets. IEEE Transactions on Visualization and Computer Graphics. 2014 Dec;20(12):1983–1992. doi:10.1109/tvcg.2014.2346248.
